# New limits of secondary β-relaxation

**DOI:** 10.1038/srep43091

**Published:** 2017-02-22

**Authors:** Satya N. Tripathy, Marzena Rams-Baron, Zaneta Wojnarowska, Justyna Knapik-Kowalczuk, Marian Paluch

**Affiliations:** 1 Institute of Physics, University of Silesia, Uniwersytecka 4, 40-007 Katowice, Poland; 2 Silesian Center for Education and Interdisciplinary Research, 75 Pulku Piechoty 1A, 41-500 Chorzow, Poland

## Abstract

Glass is an ultraviscous liquid that ceases to flow on a laboratory timescale but continues to relax on a geological timescale. Quintessentially, it has become hopeless for humans to explore the equilibrium behavior of glass, although the technology of glass making witness a remarkable advance. In this work, we propose a novel thermodynamic path to prepare a high density amorphous state of matter (carvedilol dihydrogen phosphate) using high pressure. In addition, we provide the impeccable experimental evidence of heterogeneous nature of secondary β-relaxation and probe its properties to understand the various aspects of pressure densified glass, such as dynamics, packing and disorder. These features are expected to provide new horizons to glass preparation and functional response to pharmaceutical applications.

A liquid starts to flow when its structure gets relaxed. The thermal fluctuations drive the molecules of the liquid to move constantly and establish the relaxation of the structure on short timescales. This relaxation of structure (α) is a many-body spontaneous irreversible phenomenon and directly connected to the viscosity (η) of the system^1^,^2^,^3^,^4^,^5^,^6^,^7^,^8^. When the liquid is cooled down (or compressed) fast enough to avoid the standard first-order phase transition towards equilibrium crystalline state, it enters to a metastable supercooled regime. Upon further cooling, position and dynamics of atoms are frozen by dramatic increase in dense packing, thus structural relaxation time τ_α_ becomes increasingly long. It results to a form of matter that does not flow easily on the laboratory timescale and transforms to an amorphous state of matter i.e., glass. The corresponding temperature is known as the glass transition temperature (T_g_). Thus the mystery of glass is hidden in the liquid^7^. Geometrically, the glassy state has a microscopic structure akin to a liquid but behaves mechanically like crystalline solids. Note that upon supercooling, the system explores the potential energy landscape through α-process to attain the local minimum i.e., a possible disordered packing of atoms. The depth of this minimum in which the system is trapped will depend on the thermodynamic path, because deeper the minimum, more time is required for the system to find its way to that depth^8^. The dynamics and thermodynamic properties of a glass thus depend on the process by which it is designed. Unfortunately, below T_g_ the structure of glass is not identical to the extrapolated locus of equilibrium liquid. As a result, we notice a relaxation of its structure, volume and enthalpy on geological timescale in the direction of extrapolated equilibrium values upon annealing to attain the equilibrium properties^9^. Certainly, understanding the behavior of glass (or dense amorphous state) remains an enigma in science and warrants radical treatment^10^,^11^,^12^,^13^,^14^.

Now what happens to matter near T_g_? The structural relaxation time (τ_α_), viscosity (η) and energy scale 
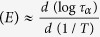
 of supercooled liquids grow in an explosive way upon cooling without change in chemical structure i.e., super-Arrhenius behaviour^15^,^16^,^17^,^18^,^19^,^20^,^21^. This experimental observation invoke that the glass formation is a cooperative phenomenon and many degrees of freedom move collectively to relax the system. The local molecular motion is not sufficient to maintain the molecular mobility at low free volume and the motion of a particular molecule is concomitant to certain degree on the neighbors. Note that close to T_g_ the structure of system lacks periodic distribution of matter, thus all atoms are not structurally equivalent and energy barrier is not a site specific quantity. It naturally suggests the appearance and enhancement of a spatio-temporal distribution of local density, energy barrier, molecular mobility (slow or fast), relaxation time (long or short) and thus the existence of cooperative dynamic length scales in the system. This growing nonhomogeneity continuum upon decreasing the temperature is a signature of dynamic heterogeneity and central to glass dynamics. Many approaches have been attempted to understand the non-exponential character and dramatic growth of structural relaxation that suggest the concept of a growing length scales^15^,^16^,^17^,^18^,^19^,^20^,^21^. *However the central issue is to understand the distribution of dynamic length scales in matter.*

Besides, when the sample is well below T_g_, structural relaxation is not accessible with in the frame of experimental time and if relaxation does occur, it tends to be more highly localized. This relaxation is known as secondary β-process that often survives the vitrification. Competing interpretations of β-relaxation are argued to understand its basic character^22^,^23^. Nevertheless β-process manifests the source of dynamics in glassy state and provides the faster time scale for attempts to escape from the trapped potential minima. *Now question arises: (a) Is secondary β-relaxation process cooperative in nature and (b) Does this process involve the coexistence of fast/slow populations of particles or modes?* To address these fundamental issues, we require a dense amorphous state of matter and a suitable probe to examine it. Note that in glassy state the perturbation in β-process is accommodated through dynamics, packing and disorder, thereby suitable tuning of β-process is expected to provide key answers to the above raised issues.

In this work, authors suggest a new thermodynamic path using high pressure to attain a high density amorphous state of matter that is anomalous to the conventional supercooling approach (path A, OG) at ambient pressure (p = 0.1 MPa). This path denotes the isothermal compression at (T_2_) of the supercooled liquid to reach the glass transition T_g_ and followed by isobaric cooling with decompression at the anticipated point of examination (T_1_) i.e., (path B, DG) as shown in schematically in Fig. 1 inset. The pressure at the examination point for both the samples is equal to p = 0.1 MPa. We investigate the conductivity and secondary relaxation time to compare the dynamic properties of glass formed by conventional and proposed path using carvedilol dihydrogen phosphate (T_g_ ≈ 341 K). We select this protic ionic glass former because it exhibits ample decoupling feature so that the conductivity relaxation time, τ_σ_ enables us to monitor the system in glassy state in experimental time frame^24^.

## Results and Discussion

**Figure 1:**
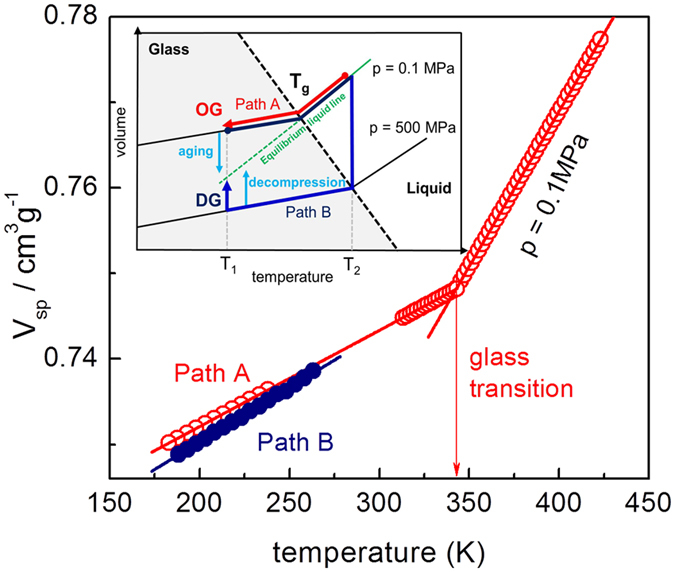
The specific volume (V_sp_) of carvedilol dihydrogen phosphate as a function of temperature using two thermodynamic paths. Inset: Schematic illustration of thermodynamic paths leading to various glassy structures (OG - ordinary glass using path A, DG - pressure densified glass using path B). T_1_ represent the temperature of examination point whereas T_2_ indicate the temperature corresponding to isothermal compression of OG to attain glassy state. The DG sample is obtained after following path B with decompression at T = T_1_. Both the samples OG and DG are compared at (T_1_, p = 0.1 MPa).

As liquid is supercooled at constant pressure, a drop in kinetic energy and rise in the density takes place. However, vitrification by isothermal compression leads to only perturbation in the density of the system^5^. As the thermodynamic variable pressure directly acts only on intermolecular distance thus we expect that different energetic modes in glassy state can be appropriately tuned^25^. In consequence, with the aim to achieve a high density glassy state, pressure driven structural reorganization is more convenient than isobaric supercooling approach. *However, what is the extent of molecular packing of a glass formed through isothermal compression and its deviation to conventional isobaric supercooling?* To verify our approach, we compare the specific volume (v_sp_) of OG and DG as a function of temperature as shown in Fig. 1. We distinguish that DG exhibit a robust change in density compared to OG in glassy state. Furthermore, the change in density magnitude increases with decreasing temperature and provide the direct experimental evidence of efficiently packed structures. Certainly, we have now attained a glassy state of matter with high density to examine and understand the property of such glass. *But how the dynamic and thermodynamic properties of this new glass differ from conventional glass?*

**Figure 2:**
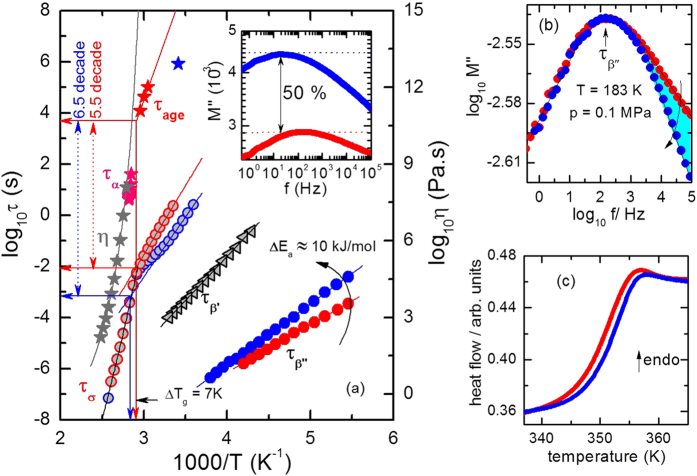
(**a**) Relaxation map of carvedilol dihydrogen phosphate. The open red and blue circles represent the conductivity relaxation time (τ_σ_) for OG and DG respectively estimated from dielectric modulus spectra. The gray and pink star symbols refer to OG sample and illustrate viscosity data (η) and structural relaxation times (τ_α_) which were determined from temperature modulated differential scanning calorimetry (TMDSC). The red and blue stars depict the aging time (τ_age_) for OG and DG respectively. The black open triangles show the temperature dependence behavior of slower secondary β^′^-relaxation times (τ_β′_) of OG. The blue and red circles present the faster secondary β^″^-relaxation times (τ_β″_) of DG and OG respectively. The inset represent the comparison plot of β^″^-spectra in dielectric modulus representation (M″) for OG and DG at T = 183 K and p = 0.1 MPa. (**b**) time temperature superposition of β″-modulus spectra at T = 183 K and p = 0.1 MPa of OG and DG (**c**) DSC traces obtained for both the paths with heating rate equals to 10 K/min. The solid blue and red lines show the behavior of DG and OG glasses, respectively. Reprinted (η and τ_α_ data) with permission from Wojnarowska, Z., Wang, Y., Pionteck, J., Grzybowska, K., Sokolov, A.P., Paluch, M. Phys. Rev. Lett. 111, 225703-225703, 2013. Copyright 2013 by the American Physical Society. https://doi.org/10.1103/PhysRevLett.111.225703 The τ_age_ values are reprinted (adapted) with permission from Wojnarowska, Z., Roland, C.M., Kolodziejczyk, K., Swiety-Pospiech, A., Grzybowska, K., Paluch, M. J. Phys. Chem. Lett., 3(10), 1238–1241, 2012. Copyright 2012 American Chemical Society.

To understand the properties (such as dynamics, packing, disorder and stability) of a densified glass, we compare the relaxation map of carvedilol dihydrogen phosphate for OG and DG in Fig. 2(a). For path A, the conductivity relaxation time 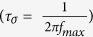 i.e., the peak maximum of imaginary part of dielectric modulus spectra M″(f), originating from the ionic motion moves toward lower frequencies upon cooling (see also Fig. S2). However, its thermal activation is reduced considerably below a certain temperature. The temperature dependence locus of conductivity relaxation times changes from Vogel-Fulcher-Tammann (VFT) like to Arrhenius behavior and physically implies the manifestation of glass transition^24^. Interestingly below T_g_, both the glasses exhibit different dynamics that is evident by change in slopes involving thermal activation of τ_σ_ with a rise in T_g_ about 7 K in DG which indicates the improved thermodynamic stability. This behavior is also translated through the drop in activation energy of σ-process for path B (E_a_ = 93.68 kJ/mol ± 0.76) compared to path A (E_a_ = 102.16 kJ/mol ± 0.4) in glassy state. We note that in DG, *τ*_*σ*_ is significantly faster than OG in glassy state which is due to the more facile proton hopping through dense hydrogen-bonded network (Grotthuss mechanism)^24^. In addition, upon supercooling the τ_σ_ for both the glass are congruent, establishing identical supercooled state and thus the thermal activation of τ_σ_ for both the glass in supercooled state are well described by same VFT^3^ relation by eq. (1)


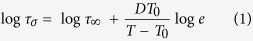


for log τ_∞_ = −17.5 s ± 0.6, D = 13.71 ± 1.48, T_0_ = 247.37 K ± 5. The log τ_∞_ denote the limiting dielectric relaxation time at the high temperatures, T_0_ denotes ideal glass transition temperature, and D is the fragility parameter. In supercooled liquid state, we compare the τ_σ_ of OG with viscosity and structural relaxation time τ_α_ data collected from refs 24, 26 in Fig. 2(a). It is found that τ_σ_ does not mimic the viscosity (η) and there exists a decoupling of 5.5 decade on time scale at T_g_. This decoupling feature is further amplified in DG by 6.5 decade. Now, in order to verify the timescale difference of τ_*σ*_ between both the glasses, we have carried out aging experiment (see Supplementary material). The time evolution of τ_σ_ of DG at T = 293 K is given by^26^


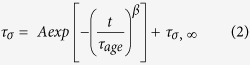


Where A, β, and τ_σ,∞_ are constants and *τ*_*age*_ is governed by the slow structural relaxation dynamics of the glass. The τ_age_ is the aging time required by the system to attain equilibrium state. It shows that due to enhanced proton conductivity in DG than OG, a time scale difference of approx. 4 years is observed upon aging. Further, comparing our result with ref. 26, we found that τ_age_ of DG falls below the extrapolated line of temperature dependent τ_age_ of OG. Thus it is clear that the DG would take less τ_age_ to attain the equilibrium state compared to OG, because of its high packing of matter.

Below T_g_, we observe two secondary relaxation processes^22^, one at low frequency (denoted as β′-process) and other at high frequency (denoted as β″-process). Both the relaxations move towards lower frequency side upon cooling and obey the Arrhenius law^3^ described by eq. (3)


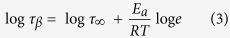


where E_a_ is the activation barrier and the log τ_∞_ denote the limiting dielectric relaxation time at the high temperatures. Here, we probe only the faster β′-process because slower β′-process is considerably masked by d.c. conductivity that influences the precise determination of the relaxation time. The estimated Arrhenius parameters for β″-process are (path A: E_a_ = 35.64 kJ/mol ± 0.64, 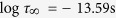) and (path B: E_a_ = 45.62 kJ/mol ± 0.27, 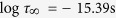). Thus ample increase in activation energy suggests that the structure of glass is strongly perturbed by thermodynamic path. From the perspective of potential energy landscapes, it is clear that basins responsible for the β″-relaxation are separated by barriers with higher height compared to OG. In other words, in compressed glass the relaxing species contributing β″-relaxation explore deep states in the basin. This increase in activation barrier is a strong evidence of structural reorganization below T_g_. Now, it is also anticipated that this observation should be reflected through the strength of dielectric M″ (f) profile. As a consequence, a rise in magnitude of M″ (f) strength by 50% (or suppression of dielectric strength) is observed which is interpreted as rise in kinetic stability as shown in Fig. 2(a)-inset. Analogous type of observation has been reported by Yu *et al*. in vapor deposited toluene^25^. Further, we note that the β″-relaxation spectra registered at thermodynamic condition (p = 0.1 MPa, T = 183 K) for DG moves nearly ∆τ_β″_  ≈ 1 decade towards low frequency compared to OG. In addition, the difference in time scale of β-process for both the glass decreases with increasing temperature. It implies more packed structure and support PVT measurement (See Fig. 1).

Remarkably, we notice ample narrowing of peak shape of β″-relaxation (increase in the slope of the high frequency wing) upon changing thermodynamic path. Figure 2(b) shows the time-temperature superposition of β″-relaxation spectra for both the glasses obtained at T = 183 K and p = 0.1 MPa. It is suggested that glasses and supercooled liquids comprise of distinct subensembles of molecules with fast or slow mobility with respect to the average relaxation rate^1^,^2^,^3^. In other words, there exists a distribution of relaxation times^27^ or molecular mobilites. Thus, the existence of slow and fast subensembles in the glassy state should also result in distinct relaxation rates for these different regions in the system to approach equilibrium state. Eventually, due to this dynamical heterogeneity, it is expected that the spectral shape of the response function of a relaxation process should perturb as a function of time. Unambiguously, Fig. 2(b) reveals that the secondary β″-relaxation entails the coexistence of slow and fast modes that contribute to the distribution of relaxation time^21^,^28^,^29^. The faster modes at high frequency in the distribution of relaxation times progress toward equilibrium quickly than the modes at lower frequency. This experimental observation supports the existence of the heterogeneous nature of secondary relaxation in the examined protic ionic glass former. Again, it also reveals the extent of dynamic heterogeneity below T_g_^29^ and less heterogeneous packing of matter. Note that in glassy state any particle may exchange its position with the neighboring one with a finite probability. Thus the relaxing units contributing β″-relaxation should be influenced by certain degree with nearby cooperative region of particles governing the structural relaxation^20^,^21^. The local atomic re-arrangement through short-range diffusion or cooperative atomic motion is manifested by the rise of the activation barrier that leads to the structure evolution and thereby the unlocking the secrets of dynamic heterogeneity in glassy state.

In order to understand the energy configuration, we have carried out calorimetric characterization for both path A and path B. As can be seen in Fig. 2(c), path B shows a suppressed nature of overshoot in heat capacity compared to path A that indicates higher free energy in the sample. At this juncture it is essential to note that compared to OG, activation barrier for β-process increases by 10 kJ/mol in DG which indicate structural evolution in glassy state. Thus the thermally activated units contributing to β-dynamics move deeper into the potential well due to increase in barrier height and it is expected that energy (or liquid like structure) is locally locked in the glass formed by path B. As a result, upon melting of DG near T_g_, the release of locked energy takes place that compensates the large heat capacity overshooting which is expected for a thousand years old glass or vapor deposited glass (VDG). This feature makes the path B unique and different from VDG because it has enhanced kinetic and thermodynamics stability with high free energy^25^. This suppression of heat capacity is also observed for other glass former such as etoricoxib.

## Conclusion

The presented herein high pressure approach is expected to provide new horizons to understand the behavior of an densified glass and intrinsic nature of heterogeneous secondary relaxations. We believe that results presented in our manuscript may be the beginning of stimulating scientific discussion on the novel approaches being attractive from the pharmaceutical viewpoint, since prepared DG glasses due to higher energy should offer beneficial water solubility, whereas same time enhanced proton conductivity may be attractive for electrochemical applications.

## Methods

Carvedilol dihydrogen phosphate was purchased from Chemical Department of Polpharma SA as crystalline powder. The first investigated material described as ordinary glass (OG) was obtained conventionally by quench-cooling of the melt (i.e. path A, see inset of Fig. 1). The second sample denoted as densified glass (DG) was produced *via* multistage route involving: (1) isothermal (T_2_ = 413 K) compression to p = 500 MPa, (2) isobaric cooling to T_1_ = 293 K, (3) isothermal (T_1_ = 293 K) decompression to p = 0.1 MPa (i.e. path B). We used herein automatic high-pressure system developed by Unipress and described in details elsewhere^30^. The capacitor with quenched sample (diameter 15 mm; gap 0.1 mm; Teflon spacer) was placed in the high-pressure chamber pre-heated to T = 413 K and proceeded as describe above. Both samples were analyzed by means of broadband dielectric spectroscopy (BDS) and differential scanning calorimetry (DSC).

To understand the relaxation properties of both systems we performed dielectric measurements in a wide range of frequencies (10^−1^ Hz to 10^6^ Hz) and temperatures (from T = 183 K to T = 373 K, ΔT = 5 K) using a Novocontrol GMBH Alfa analyzer. The temperature was precisely controlled ( ± 0.1 K) by Quatro cryosystem. The dielectric data has been analyzed in the dynamic window of dielectric modulus formalism (details have been provided in the Supplementary material).

Thermal properties of both materials were examined by means of a Mettler-Toledo DSC 1 STAR^e^ System. The measuring device was calibrated for temperature and enthalpy using zinc and indium standards, and was equipped with HSS8 ceramic sensor having 120 thermocouples and liquid nitrogen cooling accessory. The samples were measured in an aluminum crucible (40 μL). During the experiment the samples have been heated up from 293.15 K to 443.15 K with heating rate equal to 10 K/min. The glass transition temperature has been determined as the midpoint of the heat capacity increment.

## Additional Information

**How to cite this article**: Tripathy, S. N. *et al*. New limits of secondary β-relaxation. *Sci. Rep.* **7**, 43091; doi: 10.1038/srep43091 (2017).

**Publisher's note:** Springer Nature remains neutral with regard to jurisdictional claims in published maps and institutional affiliations.

## Supplementary Material

Supplementary Materials
