# Supplementary material for: New limits of secondary β-relaxation
Source: Sci Rep. 2017 Feb 22;7:43091. doi: 10.1038/srep43091 (PMC5320533; doi:10.1038/srep43091)
Supplement: Supplementary Materials [file srep43091-s1.pdf]

Supplementary Material

For

New limits of secondary *β*-relaxation

Satya N. Tripathy, M. Rams-Baron*, Z. Wojnarowska, J. Knapik and M. Paluch*

Institute of Physics, University of Silesia, Uniwersytecka 4, 40-007 Katowice, Poland.
Silesian Center for Education and Interdisciplinary Research, 75 Pulku Piechoty 1A, 41-500 Chorzow, Poland.

*author of correspondence: marzena.rams-baron@us.edu.pl, marian.paluch@us.edu.pl

Experimental Details

A. Chemical structure of carvedilol dihydrogen phosphate

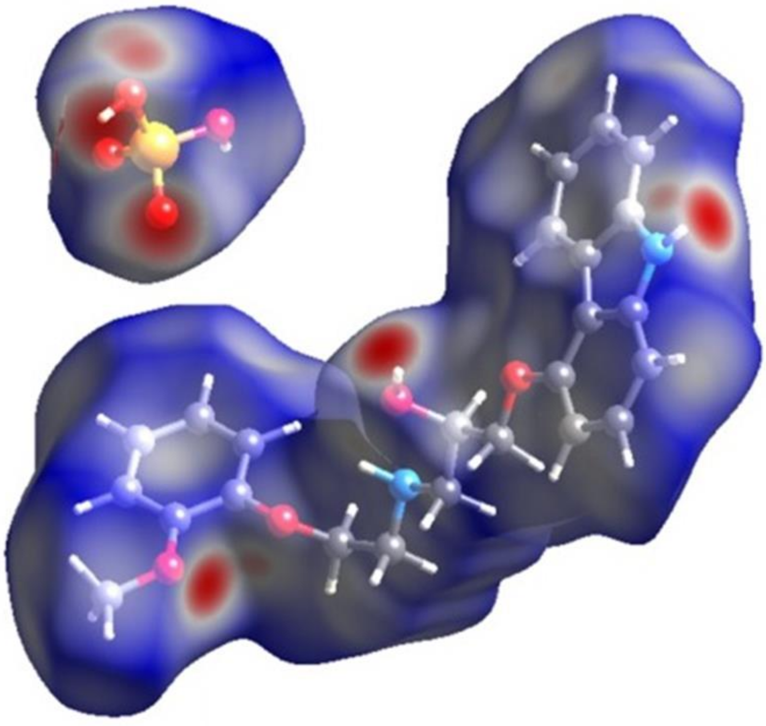

Figure S1. The chemical structure of carvedilol dihydrogen phosphate molecule. The particular colors denote: yellow – phosphorus, red – oxygen, blue – nitrogen, grey – carbon, white – hydrogen atoms, respectively.

B. The analysis of dielectric data

The dielectric data were presented and analyzed in the electric modulus representation M''(f). To determine conductivity relaxation times we have used the Havriliak-Negami function, represented mathematically by the following equation:

$$F^{*}_{HN}(\omega) = \frac{1}{[1+(i\omega\tau_{HN})^{\alpha}]^{\beta}} \quad \text{(S1)}$$

where τ_{HN} denotes the characteristic relaxation time; exponents α and β characterize symmetric and asymmetric broadenings of the peak profile and ω is an angular frequency. The relaxation times of conductivity relaxation and secondary relaxation were further estimated using the following formula:

$$\tau = \tau_{HN}\left[\sin\left(\frac{\alpha\cdot\pi}{2+2\beta}\right)\right]^{-1/\alpha}\left[\sin\left(\frac{\alpha\cdot\beta\cdot\pi}{2+2\beta}\right)\right]^{1/\alpha} \quad \text{(S2)}$$

Dielectric Results

A. Temperature dependent dielectric M″(*f*)

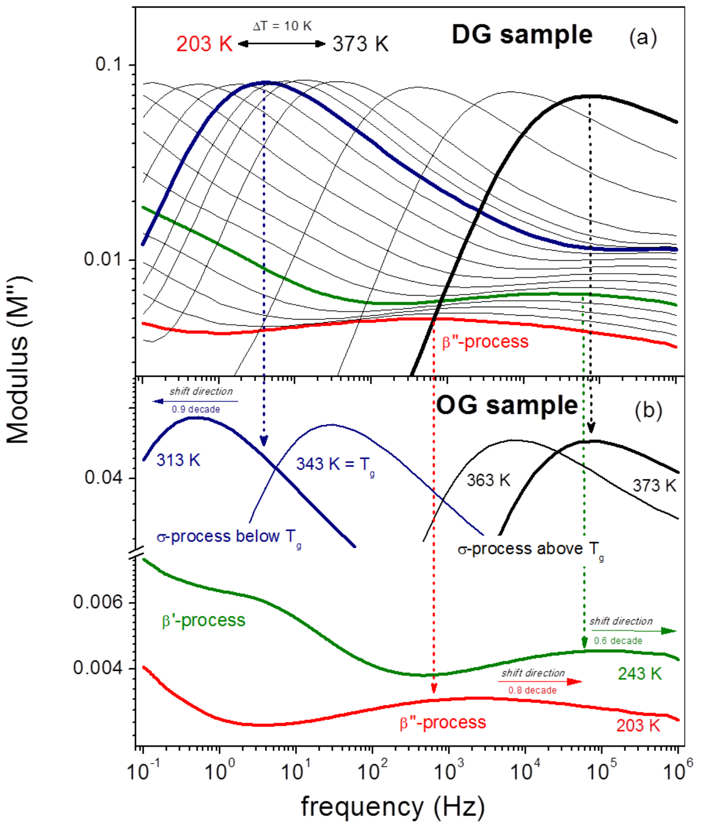

Figure S2. The imaginary part of dielectric modulus (M″) spectra registered for densified (panel A) and ordinary (panel B) samples of carvedilol dihydrogen phosphate at ambient pressure above and below the glass transition temperature. Arrows indicate the variations in peak positions observed for DG and OG samples both in supercooled liquid and glassy state.

Figure S2 (a) and (b) depict the temperature dependent behavior of imaginary part of dielectric modulus M″(f) for both OG and DG sample measured over seven decades of frequency (f = 10^{-1} - 10^{6} Hz) in a wide temperature range (203 K - 373 K). In the experimental window of M″(f) we noticed the existence of three relaxation processes: (i) the spectra recorded from 203 K to 313 K display two well defined secondary relaxations, corresponding to some localized motions that move toward high frequencies with increasing temperature for both the glass formers, (ii) then asymmetric conductivity relaxation peak initating from the ion transport becomes the central feature in the modulus spectra which moves towards high frequency side with increasing temperature. Mathematically the dielectric modulus $M^*(\upsilon)$ is represented by below equation[1-3]

$$M^*(f) = \frac{1}{\varepsilon^*(f)} = \frac{i2\pi f \epsilon_0}{\sigma^*(f)} \qquad \text{(S3)}$$

where $\varepsilon^*(\upsilon)$ is the dielectric loss function, ϵ_0 is the permittivity of vacuum and f corresponds to frequency. For ionic conductors the complex electric modulus $M^*(\upsilon)$ approach is the most appropriate for data presentation. $M^*(\upsilon)$ formalism signifies ion conductivity relaxation which

differs from dipole relaxation in supercooled liquids. Physically the electric modulus relates to the relaxation of the electric field **E** in the material when the electric displacement vector **D** remains constant *i.e.*, **E**(t) = **E**(0)ϕ(t). ϕ (t) is the relaxation function, by the following Kohlrausch-Williams-Watts (KWW) function[1-3]

$$\phi_{KWW} = exp\left[-\left(\frac{t}{\tau_\sigma}\right)^{1-n}\right] \qquad \text{(S4)}$$

Where 0 < n < 1, in the exponent is the measure of interaction and correlation and τ_σ is the conductivity relaxation time.

B. Physical Aging experiment at T = 293 K

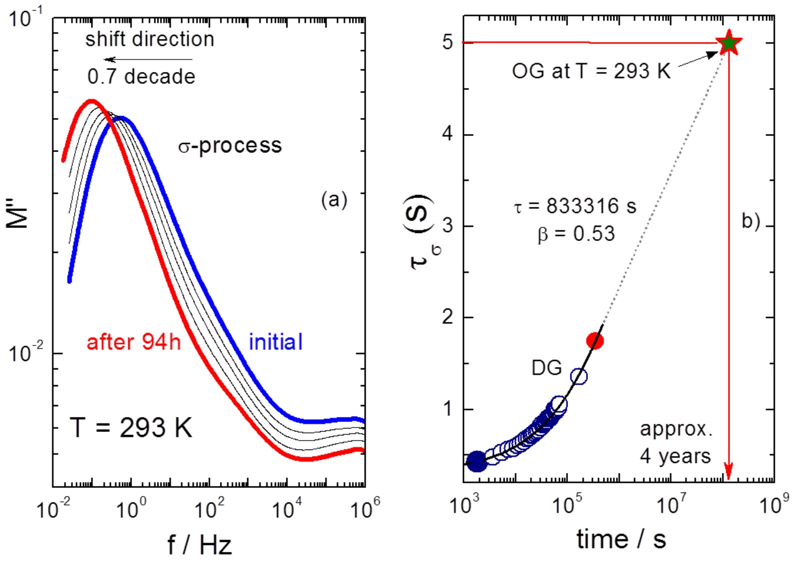

Figure S3. (a) The dielectric modulus spectra M″(f) registered during aging of DG sample at T = 293 K. The arrow indicates the shifting of conductivity relaxation peak towards lower frequency with time of about 0.7 decade. (b) Time evolution of conductivity relaxation times described by stretched exponential function (eq. S5) with fitting parameters τ_{age} = 833316 s and β =0.53.

Figure S3 shows the time-dependent behavior of the modulus spectra at T = 293 K with aging time ranging from t = 0 s to t = 94 hours. It is noticed that the conductivity relaxation times (τ_σ) moves toward low frequency side upon aging establishing the slowing down of ion-dynamics. The time evolution of τ_σ of DG at T = 293 K is given by[1-3]:

$$\tau_\sigma = Aexp\left[-\left(\frac{t}{\tau_{age}}\right)^{\beta}\right] + \tau_{\sigma,\infty} \qquad \text{(S5)}$$

where A, β, and $\tau_{\sigma,\infty}$ are constants and τ_{age} is governed by the slow structural relaxation dynamics of the glass. Time evolution of conductivity relaxation can be described by stretched exponential function described elsewhere with τ_{age} = 833316 s and β =0.53. In addition, it can be seen that the difference exists in an aging time scale of approx. 4 years between DG and OG.

C. Nature of Secondary Relaxations

Below T_g, we observed two secondary processes, one at low frequency (denoted as β′-process) and other at high frequency (denoted as β″-process). It has been well established that there are two possible molecular origins of secondary relaxation phenomena[4-5]. The former one is associated with molecular internal degrees of freedom (i.e., intra-molecular). But, the latter one originates from local motions of the entire molecule (i.e., inter-molecular; a special case of a rigid unit) which falls into the special class of Johari-Goldstein (JG) process[4].

A remarkable empirical relation of the JG secondary β-relaxation to the glass transition temperature T_g of glass-formers given by $E_\beta \approx 24\ RT_g$ where R is the gas constant, was noted by Kudlik et. al. and valid for many glass formers. In the present glass former, we found that the slower β′-relaxation exhibit $E_{\beta'} \approx 23.3\ RT_g$[4-5]. This experimental observations support the evidence of the JG nature of β′ relaxation. In the case of a rigid glass former, the absence of intramolecular degrees of freedom guarantees that slower secondary relaxation is a JG process, by its definition of involving the motion of entire molecule. The pre-exponential factor (τ_∞) of the temperature dependence of the characteristic times of the order 10^{-15} s, agrees with the ones usually observed in JG processes.

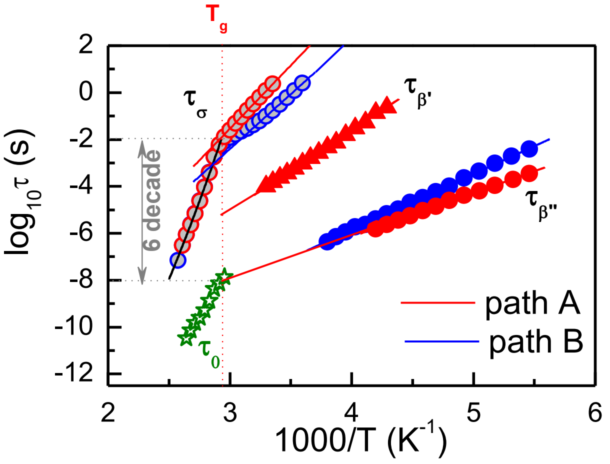

Figure S4: Relaxation map of the carvedilol dihydrogen phosphate at ambient pressure above and below the glass transition temperature. τ_0 is a precursor of primary conductivity relaxation.

Now we employ Ngai coupling model (CM)[4-5] to understand the possible connection of β″ -process to the JG relaxation and the mathematical relation is given by $\tau_0 = (t_c)^n\ (\tau_\sigma)^{1-n}$. Here, the exponent (n) is the measure of interaction. The τ_0 is the primitive conductivity relaxation time of the coupling model and t_c is the crossover time from independent relaxation to the cooperative relaxation and equals to ~ 2ps for molecular liquids. It is well recognized that the independent relaxation time τ_0 is approximately located near the most probable relaxation time $\tau_{\beta''}$ of the JG relaxation at all values of temperature. *i.e.,* $\tau_0\ (T) \approx\ \tau_{\beta''}\ (T)$. In relaxation map, we found a connection between the τ_0 to the faster relaxation process $\tau_{\beta''}$ (extrapolated) as shown in Fig.S4.

Since τ_0 is a precursor of primary conductivity relaxation, involves macroscopic ion transport and intermolecular in nature, we expect that β″-process carries similar origin. Thus we believe that the fast β″- relaxation may be due to ionic nature of JG kind[4-5]. Again the CM model connects the ratio of the conductivity relaxation and JG relaxation to the breadth of the conductivity relaxation and mathematically represented as $\left(\log \tau_{\sigma} - \log \tau_{\beta''}\right) \approx n(\log \tau_{\sigma} - \log t_c)$. This equation also has been verified for large number of glass formers and is consistent with our dielectric data. Performing the simple calculation of σ-process at T_g = 341 K and n = 0.6 (from fitting analysis), we found that $\left(\log \tau_{\sigma} - \log \tau_{\beta''}\right) = 6\ decade$ and $(\log \tau_{\sigma} - \log t_c) = 10\ decade$ in frequency. Besides the pre-exponential factor (τ_{∞}) in Arrhenius equation have values of the order 10^{-13} - 10^{-15} s, which agrees with the ones usually observed in JG processes.

D. Description of procedure applied to determine specific volume V_{sp} values for DG sample

To provide specific volume V_{sp} values for DG sample, we use pressure-volume-temperature (PVT) data reported previously for ordinary carvedilol dihydrogen phosphate[3]. To parametrize $V_{sp}(T)$ data points above and below T_g we applied two separate Tait equations. Obtained fitting parameters were then used to determine V_{sp} values at p = 0.1 MPa for ordinary sample. Since we observed significant shifting in β″-relaxation times for samples obtained via different thermodynamic paths we based our further calculations on assumption that the position of β″-relaxation peak may correlate with sample density. From Arrhenius-like temperature dependence of β″-relaxation times measured for ordinary sample (Figure 1). we found appropriate temperature values corresponding to β″-relaxation times observed for densified material. The specific volume values corresponding to these temperatures were then assigned to temperatures corresponding to β″-relaxation times observed for densified high energy sample. The rough $V_{sp}(T)$ values estimated for DG sample of carvedilol dihydrogen phosphate are presented as blue circles in Figure 1.

References

1. Wojnarowska et. al., J. Chem. Phys. 140, 174502 (2014).
2. Paluch et. al., PRL 110, 015702 (2013).
3. Wojnarowska et. al., PRL 111, 225703 (2013).
4. Ngai et. al. J. Chem. Phys. 120, 857-873 (2004).
5. Tripathy et. al. J. Chem. Phys. 142, 184504 (2015)
